# Supplementary material for: Method for Independent Estimation of the False Localization Rate for Phosphoproteomics
Source: J Proteome Res. 2022 May 31;21(7):1603–15. doi: 10.1021/acs.jproteome.1c00827 (PMC9251759; doi:10.1021/acs.jproteome.1c00827)
Supplement: Supplementary file 1 — pr1c00827_si_001.pdf [file pr1c00827_si_001.pdf]

A method for independent estimation of false localisation rate for phosphoproteomics

*Kerry A Ramsbottom[1], Ananth Prakash[2], Yasset Perez Riverol[2], Oscar Martin Camacho[1], Maria-Jesus Martin[2], Juan Antonio Vizcaíno[2], Eric W Deutsch[3] and Andrew R Jones[1]**

[1]Institute of Systems, Molecular and Integrative Biology, University of Liverpool, Liverpool, L69 3BX, United Kingdom
[2]European Molecular Biology Laboratory, EMBL-European Bioinformatics Institute (EMBL-EBI), Hinxton, Cambridge, CB10 1SD, United Kingdom.
[3]Institute for Systems Biology, Seattle, Washington 98109, United States
* Andrew.Jones@liverpool.ac.uk

Supplementary Table 1: Search parameters for each data set, using PEAKS, MaxQuant and Mascot/ptmRS pipelines. *PEAKS only, **MaxQuant only.

Supplementary Figure 1: log10 PTM probability vs PSM probability. a)PXD008355 (Arabidopsis data set) R2=0.00740, b)PXD000612 (Human dataset) R2=0.0132

Supplementary Figure 2: Boxplots of PSMs supporting targets and decoys with final probability >0.95 (pSTYA, 1%FDR). a) PXD008355 (*Arabidopsis* data set); b) PXD000612 (Human data set).

Supplementary Figure 3: Comparison of pX FLR estimation searching PXD007058 (Synthetic data set) using different pipelines: TPP, PEAKS, MaxQuant and Mascot/ptmRS (PD). PSMs are ordered by PTM probability (Fully tryptic, 1 % FDR). a) pAla decoy; b) pLeu decoy.

Supplementary Table 2: Comparison of pAla/pLeu Decoy FLR estimation searching PXD007058 (Synthetic data set) using different pipelines: TPP, PEAKS, MaxQuant and Mascot/ptmRS (PD). PSMs are ordered by PTM probability (fully tryptic, 1% FDR).

Supplementary Figure 4: Comparison of minimum distance between phosphorylated STY and the nearest target amino acid (Ala, Leu, Gly, Asp, Glu and Pro), compared to the STY distribution, searching PXD007058 (Synthetic data set).

Supplementary Figure 5: Comparison of FLR estimation searching PXD000138 (Synthetic data set) using localisation on different decoy amino acids: pAla, pGly, pLeu, pAsy, pGlu and pPro (TPP, fully tryptic, 1% FDR); b-g) Comparison of FLR estimation methods searching PXD007058 for each of the different decoy amino acids (TPP, fully tryptic, 1% FDR). X-axis = count of sites, y-axis is global FLR estimated as q-values.

Supplementary Table 3: Counts at pX FLR (calculated by the decoy method) for 1%, 5% and 10% using each FLR method, searching PXD000138 (Synthetic data set) (TPP, fully tryptic, 1% FDR).

Supplementary Figure 6: Comparison of minimum distance between phosphorylated STY and the nearest target amino acid (Ala, Leu, Gly, Asp, Glu and Pro), compared to the STY distribution, searching PXD000138 (Synthetic data set).

Supplementary Figure 7: Comparison of FLR estimation searching PXD008355 (*Arabidopsis* data set) using different decoy amino acids: pAla, pGly, pLeu, pAsp, pGlu and pPro (TPP, fully tryptic, 1 %FDR). a) all PSMs; b) FLR ≤ 0.05; c) all PSMs with "no-choice" hits removed; d) FLR ≤ 0.05 with "no-choice" hits removed. Figures a & b are shown in the main body of the manuscript and are repeated here for comparison.

Supplementary Table 4: Counts of sites at pX Decoy FLR for 1%, 5% and 10% threshold using each decoy amino acid: pAla, pGly, pLeu, pAsp, pGlu and pPro, searching PXD008355 (*Arabidopsis* data set) with "no-choice" hits removed (fully tryptic, 1% FDR).

Supplementary Figure 8: Comparison of pX Decoy FLR and Model FLR estimation searching PXD008355 (*Arabidopsis* data set) for pAla, pLeu, pGly, pAsp, pGlu and pPro (Fully tryptic, 1% FDR).

Supplementary Table 5: Comparison of amino acid frequency ratios between STY and the decoy amino acid for the identified peptides, identified phosphopeptides and the search database

Supplementary Figure 9: Comparison of a) pAla; b) pLeu and c) pGly Decoy FLR estimation searching PXD008355 (*Arabidopsis* data set) using different pipelines: TPP, PEAKS, MaxQuant, Mascot/ptmRS (PD) (Fully tryptic, 1% FDR).

Supplementary Table 6: Comparison of pAla/pLeu/pGly Decoy FLR site counts searching PXD008355 (*Arabidopsis* data set) using different pipelines: TPP, PEAKS, MaxQuant and Mascot/ptmRS (PD) (fully tryptic, 1% FDR).

Supplementary Figure 10: Comparison of pAla Decoy FLR estimation searching PXD008355 (*Arabidopsis* data set) using different pipelines: TPP, PEAKS, MaxQuant, Mascot/ptmRS (PD). a) ordered by combined probability; b) ordered by PTM probability (Fully tryptic, 1% FDR).

Supplementary Table 7: Counts of sites at pAla Decoy FLR for 1%, 5% and 10% using each pipeline, searching PXD008355 (*Arabidopsis* data set) (fully tryptic, 1% FDR).

Supplementary Figure 11: Comparison of pX Decoy FLR and Model FLR estimation searching PXD000612 (Human data set) for pAla, pLeu, pGly, pAsp, pGlu and pPro (Fully tryptic, 1% FDR), using TPP.

Supplementary Figure 12: Comparison of minimum distance between phosphorylated S, T or Y and the nearest target amino acid (Ala, Leu, Gly, Asp, Glu and Pro), compared to the STY distribution, searching PXD000612 (Human data set).

Supplementary Figure 13: Comparison of averaged final site probabilities for all peptides (final probability ≥0.68), split by amino acid in the +1 positions for the PXD008355 (*Arabidopsis* data set). a) STY (no decoy); b) STY with Ala decoy; c) STY with Leu decoy and d) STY Gly decoy.

Supplementary Figure 14: Comparison of averaged final site probabilities for all peptides final probability ≥0.68), split by amino acid in the -1 positions for the PXD000612 (Human data set). a) STY (no decoy); b) STY with Ala decoy; c) STY with Leu decoy and d) STY Gly decoy.

Supplementary Figure 15: Comparison of averaged final site probabilities for all peptides (final probability ≥0.68), split by amino acid in the +1 positions for the PXD000612 (Human data set) a) STY (no decoy); b) STY with Ala decoy; c) STY with Leu decoy and d) STY Gly decoy.

.

*Supplementary Table 1: Search parameters for each data set, using PEAKS, MaxQuant and Mascot/ptmRS pipelines. *PEAKS only, **MaxQuant only.*

	**PXD007058 (Synthetic data set)**	**PXD008355 (*Arabidopsis* data set)**
**Peptide Mass Tolerance**	20.0 ppm	10 ppm
**Fragment Bin Tolerance**	0.02 Da	0.02 Da
**Digest Mode**	Tryptic	Tryptic
**Max Missed Cleavages**	2 (4*)	2
**Fixed Mods**	Carbamidomethylation (C)	Carbamidomethylation (C)
**Variable Mods**	Oxidation (MWP) (Oxidation (M)*)<br>Phospho (STYX)<br>Pyrophospho (STY),<br>N-terminal acetylation**<br>Ammonia loss (QC)**<br>Pyro-Glu (EQ on the N-terminus)**<br>Deamination (NQ)**	Oxidation (M)<br>Phospho (STYX)<br>N-terminal acetylation<br>Ammonia loss (QC)<br>Pyro-Glu (EQ on the N-terminus)<br>Deamination (NQ)
**Max Variable PTMs**	3 (5*)	3

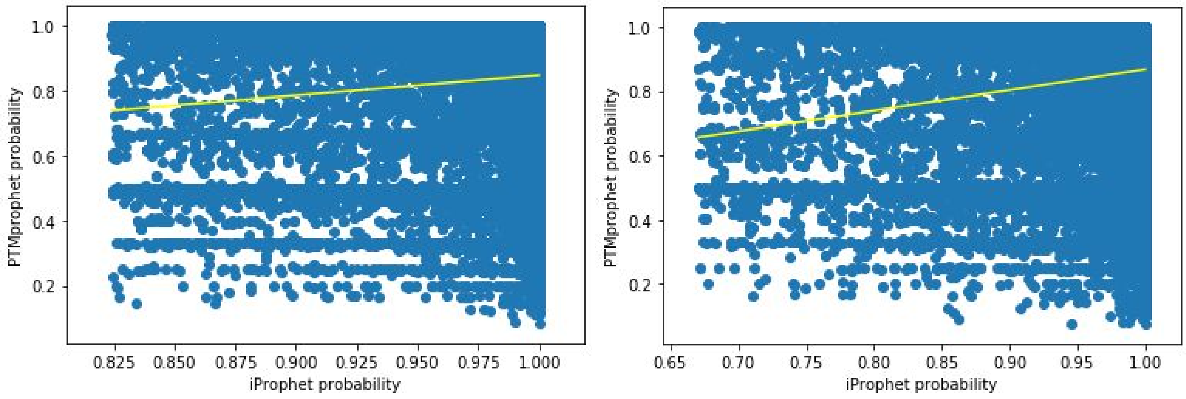

Supplementary Figure 1: log10 PTM probability vs PSM probability. a)PXD008355 (Arabidopsis data set) R2=0.00740, b)PXD000612 (Human dataset) R2=0.0132

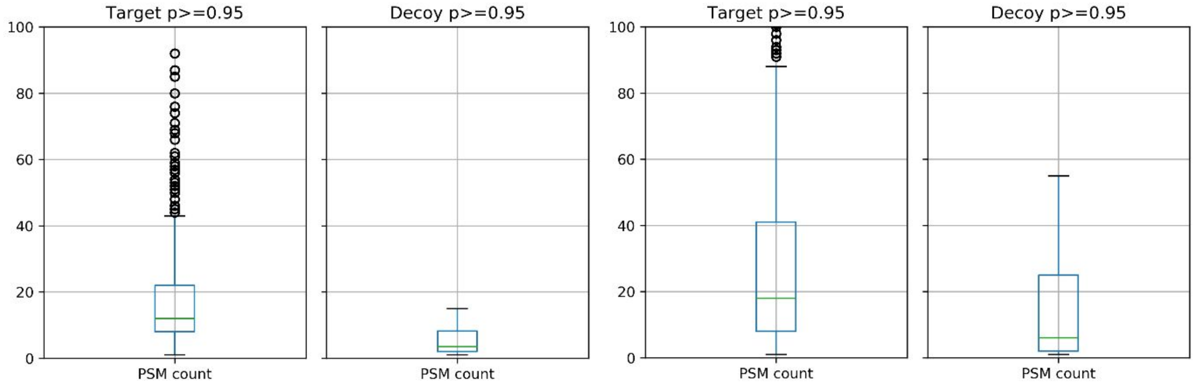

Supplementary Figure 2: Boxplots of PSMs supporting targets and decoys with final probability >0.95 (pSTYA, 1%FDR). a) PXD008355 (Arabidopsis data set); b) PXD000612 (Human data set).

a)

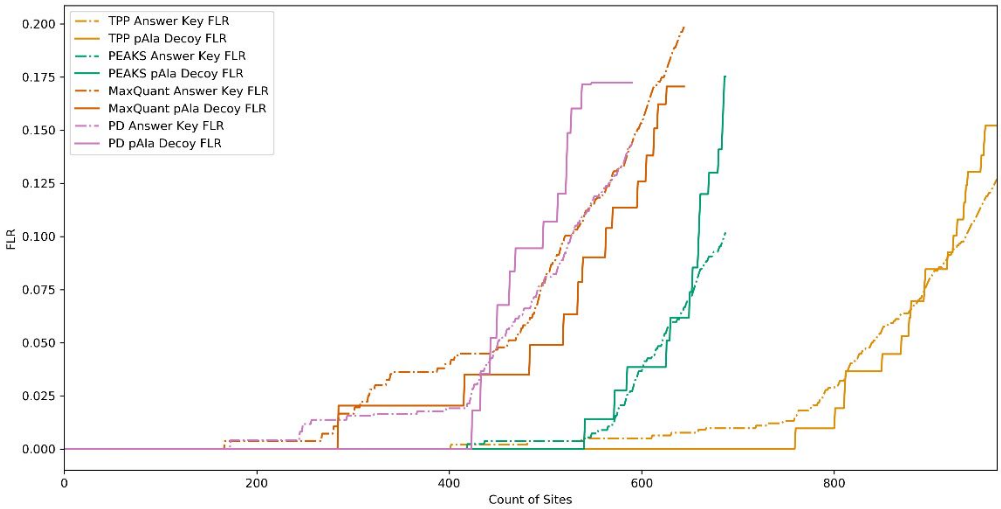

b)

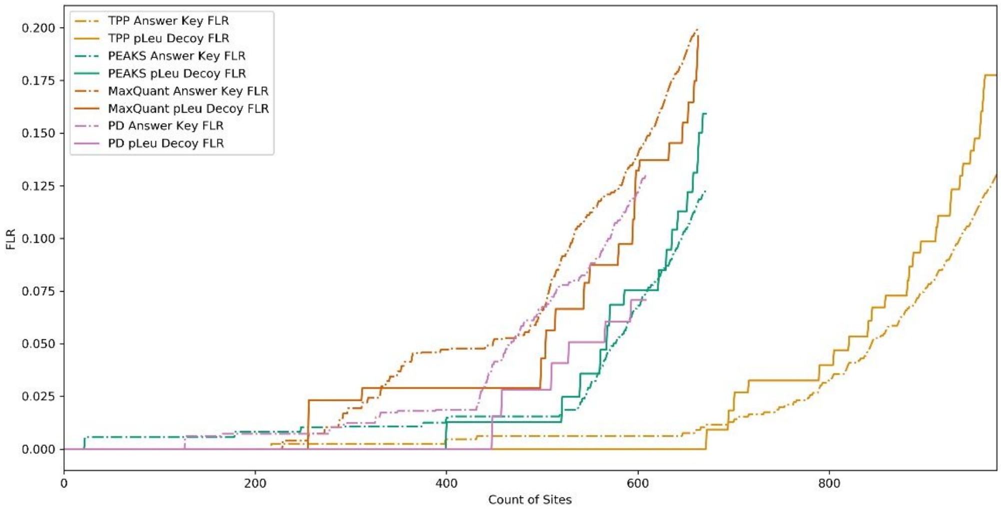

Supplementary Figure 3: Comparison of pX FLR estimation searching PXD007058 (Synthetic data set) using different pipelines: TPP, PEAKS, MaxQuant and Mascot/ptmRS (PD). PSMs are ordered by PTM probability (Fully tryptic, 1 % FDR). a) pAla decoy; b) pLeu decoy.

Supplementary Table 2: Comparison of pAla/pLeu Decoy FLR estimation searching PXD007058 (Synthetic data set) using different pipelines: TPP, PEAKS, MaxQuant and Mascot/ptmRS (PD). PSMs are ordered by PTM probability (fully tryptic, 1% FDR).

	**Count at 1% FLR**		**Count at 5% FLR**		**Count at 10% FLR**	
	**pAla**	**pLeu**	**pAla**	**pLeu**	**pAla**	**pLeu**
**TPP**	801	695	870	821	924	912
**PEAKS**	541	400	626	568	660	636
**MaxQuant**	285	256	519	504	563	595
**Mascot/ptmRS**	424	448	443	528	498	609

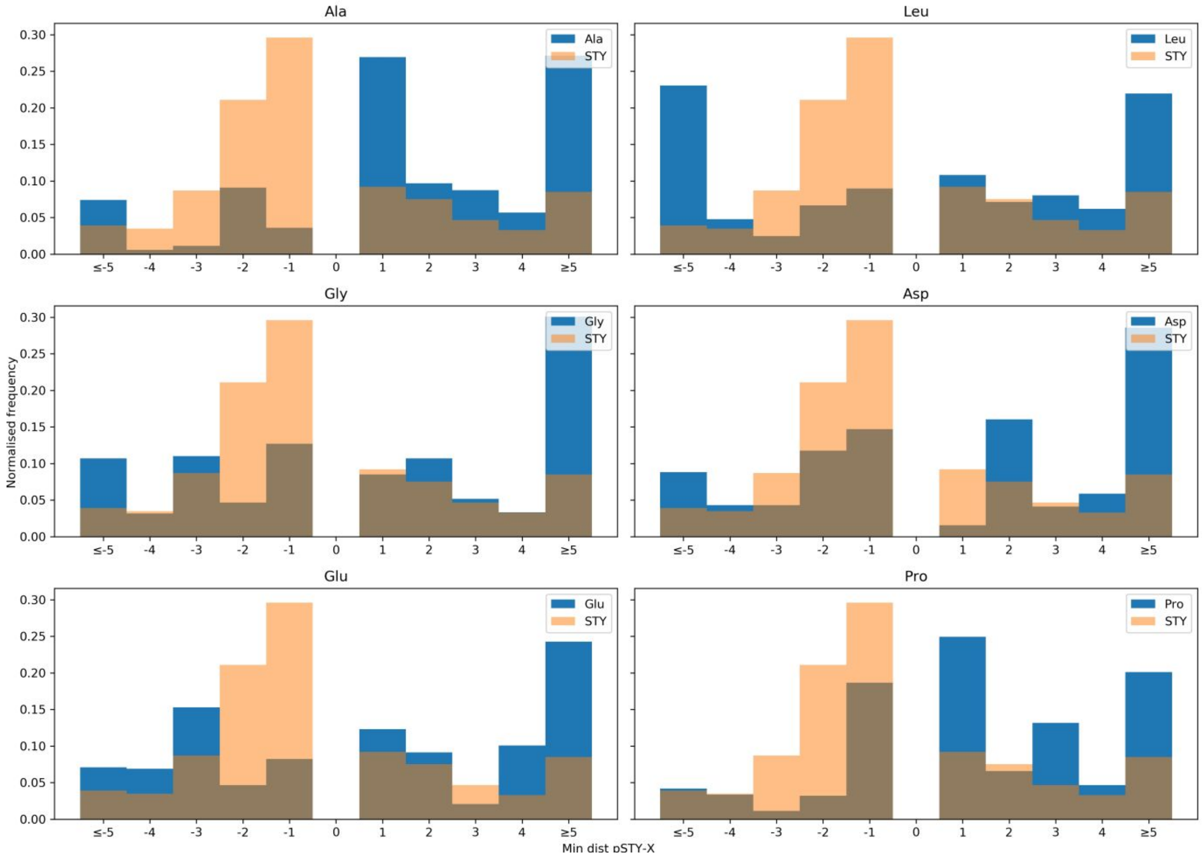

Supplementary Figure 4: Comparison of minimum distance between phosphorylated STY and the nearest target amino acid (Ala, Leu, Gly, Asp, Glu and Pro), compared to the STY distribution, searching PXD007058 (Synthetic data set).

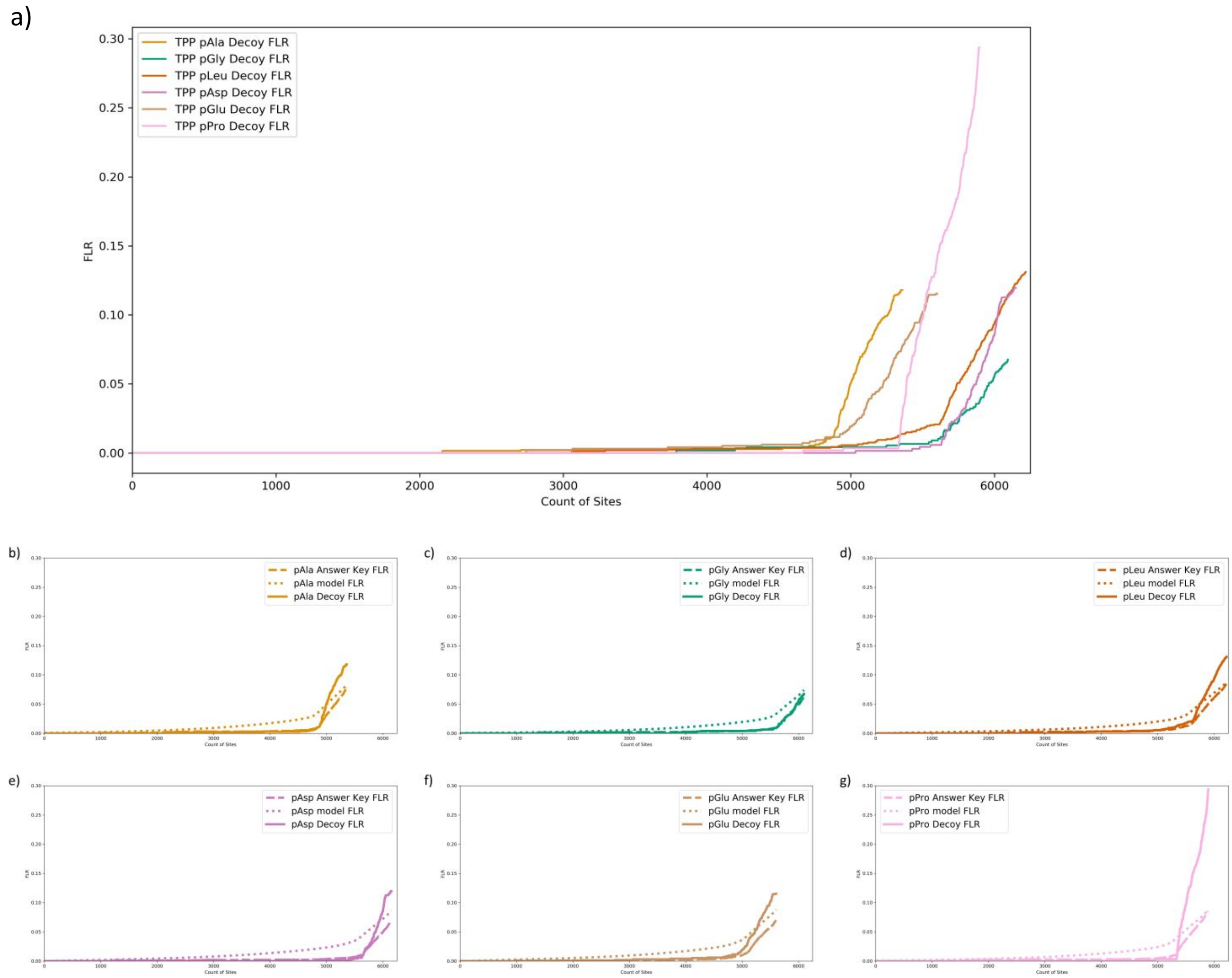

Supplementary Figure 5: Comparison of FLR estimation searching PXD000138 (Synthetic data set) using localisation on different decoy amino acids: pAla, pGly, pLeu, pAsy, pGlu and pPro (TPP, fully tryptic, 1% FDR); b-g) Comparison of FLR estimation methods searching PXD007058 for each of the different decoy amino acids (TPP, fully tryptic, 1% FDR). X-axis = count of sites, y-axis is global FLR estimated as q-values.

Supplementary Table 3: Counts at pX FLR (calculated by the decoy method) for 1%, 5% and 10% using each FLR method, searching PXD000138 (Synthetic data set) (TPP, fully tryptic, 1% FDR).

	**Count at 1% FLR**			**Count at 5% FLR**			**Count at 10% FLR**		
	**Answer Key**	**Model**	**Decoy**	**Answer Key**	**Model**	**Decoy**	**Answer Key**	**Model**	**Decoy**
**pAla**	4800	3117	4830	5116	5004	4999	5322	5360	5258
**pGly**	5573	3905	5607	5970	5810	5961	6052	6094	6094
**pLeu**	5363	3819	5289	5853	5743	5740	6175	6218	6028
**pAsp**	5582	3452	5636	5968	5714	5873	6111	6150	6023
**pGlu**	4961	3090	4818	5370	5169	5226	5564	5603	5491
**pPro**	5260	3585	5341	5579	5456	5388	5858	5896	5507

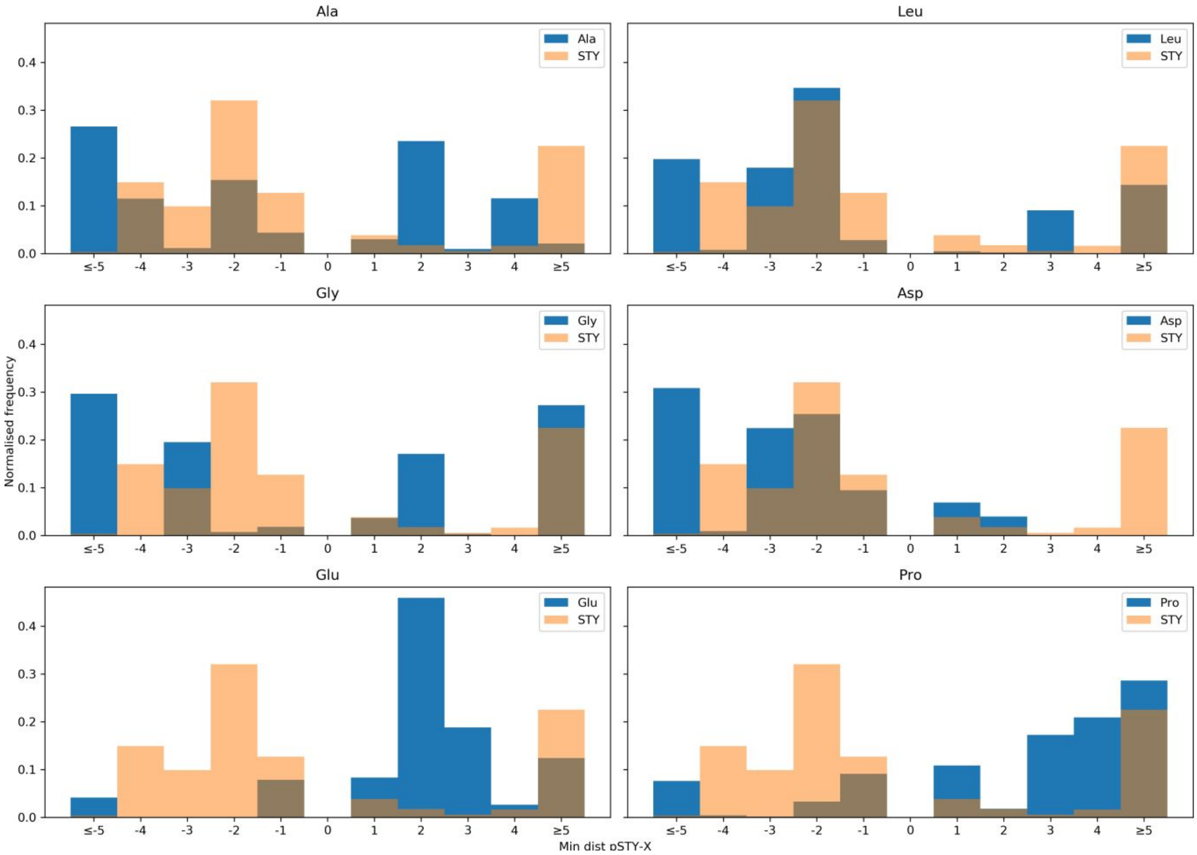

Supplementary Figure 6: Comparison of minimum distance between phosphorylated STY and the nearest target amino acid (Ala, Leu, Gly, Asp, Glu and Pro), compared to the STY distribution, searching PXD000138 (Synthetic data set).

i) Investigating high-scoring false hits

When searching the PXD008355 *Arabidopsis* database with TPP using different decoy amino acids, multiple high scoring false localisations could be seen. When these were investigated further, it was found that these wrong hits contained the same number of potential phosphosites as identified phosphosites. These wrong hits can therefore be categorised as "no-choice" PSMs as there is no choice for localisation, and thus they are wrong because the search engine result is incorrect, not the site localisation algorithm. This may indicate that the search engine and PSM scoring is producing over-confident estimates of probability. These "no-choice" hits were removed and the FLR estimations recalculated, resulting in an improvement in the FLR estimations for each method.

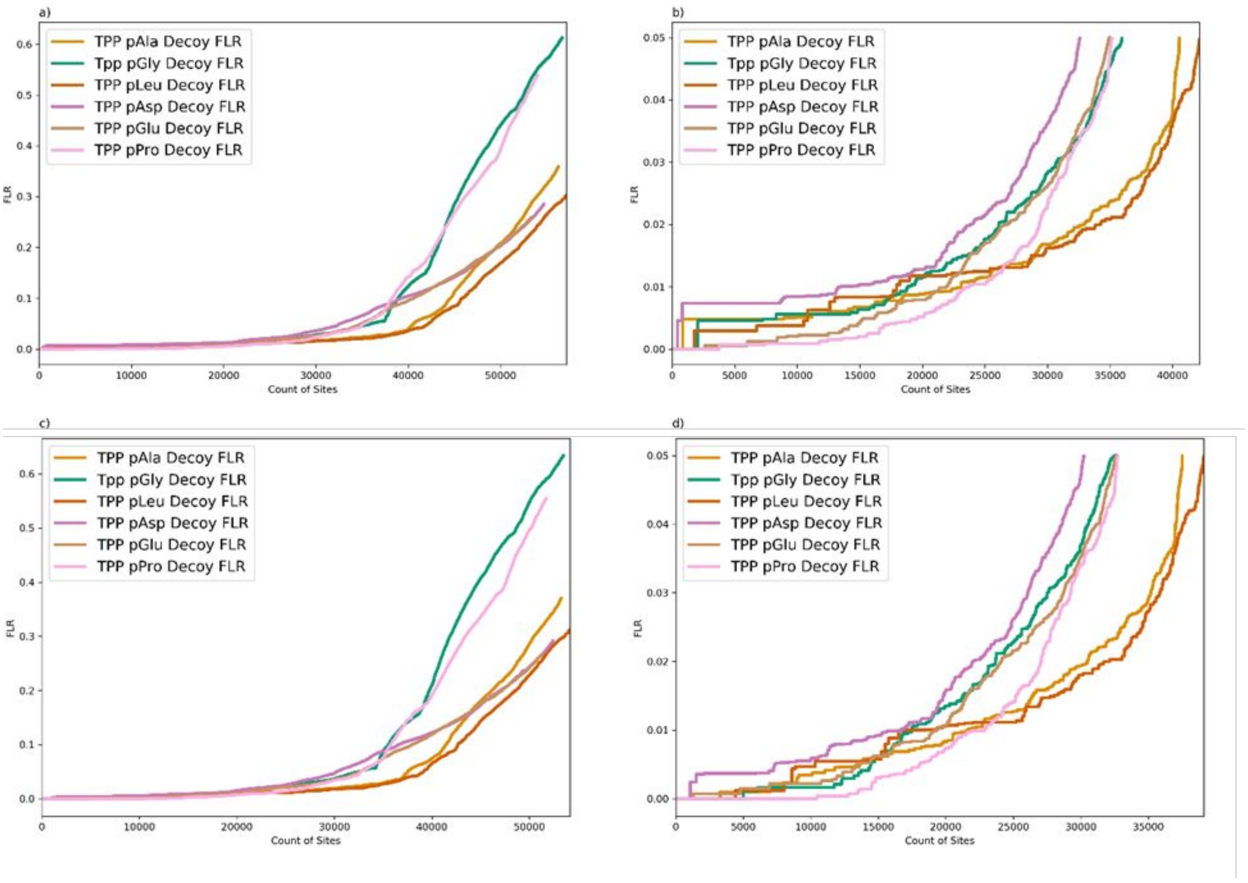

Supplementary Figure 7: Comparison of FLR estimation searching PXD008355 (Arabidopsis data set) using different decoy amino acids: pAla, pGly, pLeu, pAsp, pGlu and pPro (TPP, fully tryptic, 1 %FDR). a) all PSMs; b) FLR ≤ 0.05; c) all PSMs with "no-choice" hits removed; d) FLR ≤ 0.05 with "no-choice" hits removed. Figures a & b are shown in the main body of the manuscript and are repeated here for comparison.

Supplementary Table 4: Counts of sites at pX Decoy FLR for 1%, 5% and 10% threshold using each decoy amino acid: pAla, pGly, pLeu, pAsp, pGlu and pPro, searching PXD008355 (Arabidopsis data set) with "no-choice" hits removed (fully tryptic, 1% FDR).

	**Count at 1% FLR**	**Count at 5% FLR**	**Count at 10% FLR**
**pAla**	21689	37502	41411
**pGly**	17459	32570	35515
**pLeu**	16681	39118	42650
**pAsp**	16714	30236	36566
**pGlu**	19331	32584	37963
**pPro**	22839	32716	35743

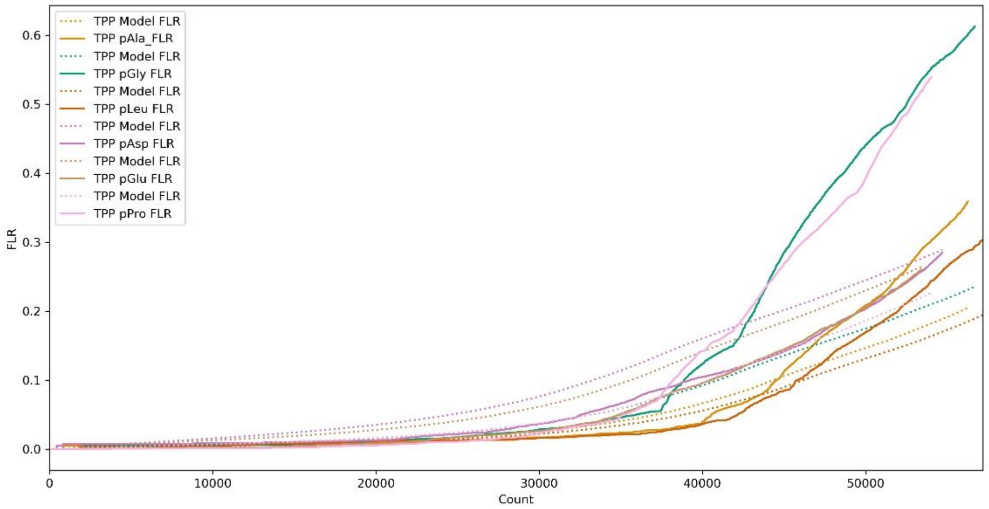

Supplementary Figure 8: Comparison of pX Decoy FLR and Model FLR estimation searching PXD008355 (Arabidopsis data set) for pAla, pLeu, pGly, pAsp, pGlu and pPro (Fully tryptic, 1% FDR).

ii) Amino acid frequency analysis

In order to try to determine the cause of the differences seen with glycine, the amino acid frequencies of the decoys used were compared across the identified peptides, phosphopeptides and search databases. It could be seen that the frequencies across peptides and phosphopeptides of Ala and Gly, compared to STY were similar across the data sets. Although differences were seen between the search databases and peptide/phosphopeptide frequencies, the database frequencies showed fairly similar frequencies across all decoy amino acids. The analysis of the amino acid frequencies was therefore unable to determine an explanation for Gly being seen as an outlier.

Supplementary Table 5: Comparison of amino acid frequency ratios between STY and the decoy amino acid for the identified peptides, identified phosphopeptides and the search database

	**PXD007058 (Synthetic data set)**			**PXD008355 *(Arabidopsis data set)***			**PXD000612 (Human data set)**		
	**Peptides STY:X**	**Phospho peptides STY:X**	**Database STY:X**	**Peptides STY:X**	**Phospho peptides STY:X**	**Database STY:X**	**Peptides STY:X**	**Phospho peptides STY:X**	**Database STY:X**
**Ala**	3.67	3.57	4.22	2.57	3.49	2.72	3.04	3.32	2.33
**Gly**	3.74	3.88	3.66	2.40	3.12	1.79	2.98	3.36	1.64
**Leu**	3.40	3.37	3.45	3.29	4.55	2.68	4.30	4.39	2.49
**Asp**	4.23	4.20	5.74	1.60	1.88	3.14	2.90	2.87	3.45
**Glu**	2.42	2.44	3.62	1.35	1.74	2.50	2.05	2.07	2.30
**Pro**	1.82	1.84	2.45	2.89	2.60	3.58	2.02	1.98	2.59

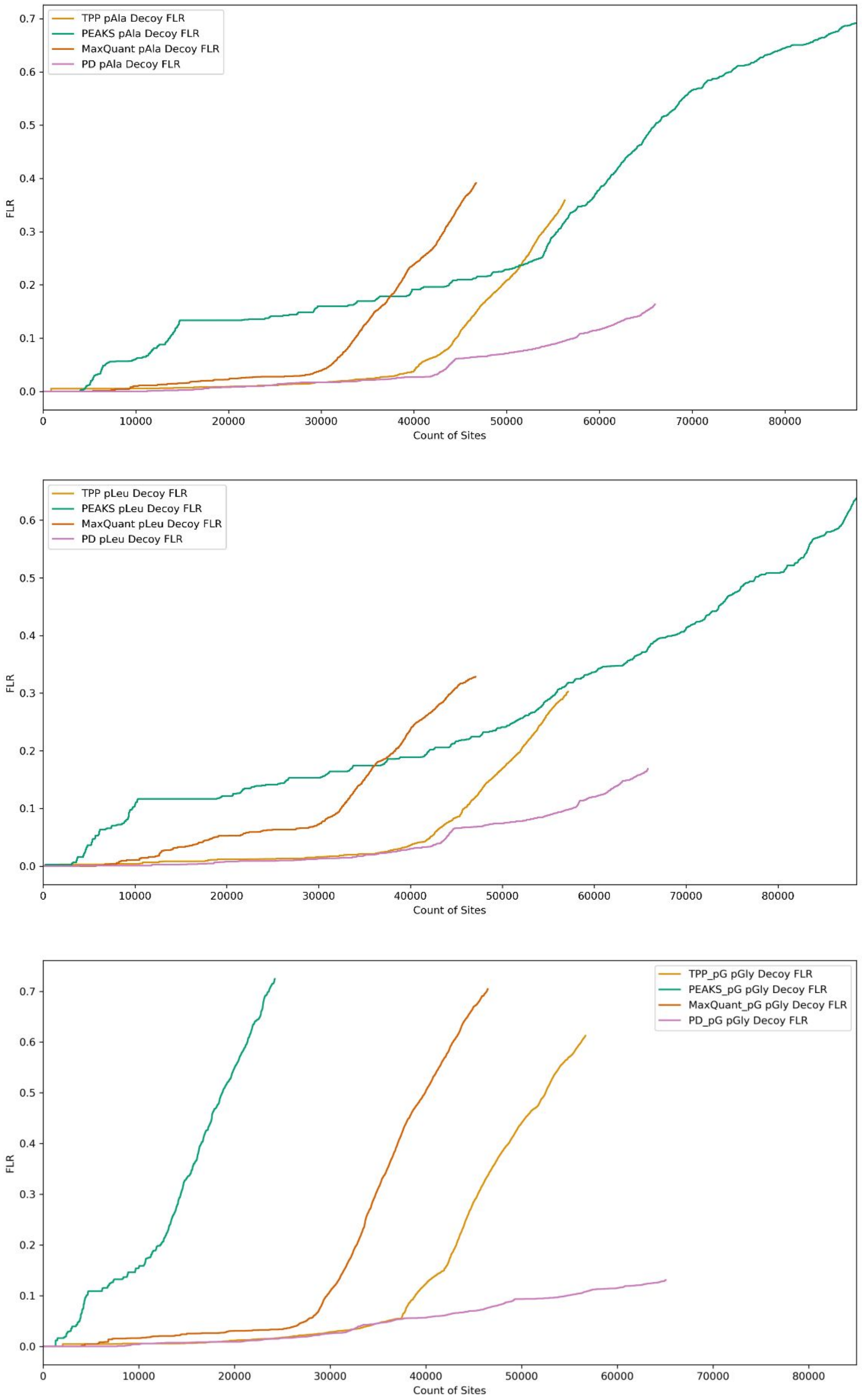

Supplementary Figure 9: Comparison of a) pAla; b) pLeu and c) pGly Decoy FLR estimation searching PXD008355 (Arabidopsis data set) using different pipelines: TPP, PEAKS, MaxQuant, Mascot/ptmRS (PD) (Fully tryptic, 1% FDR).

Supplementary Table 6: Comparison of pAla/pLeu/pGly Decoy FLR site counts searching PXD008355 (Arabidopsis data set) using different pipelines: TPP, PEAKS, MaxQuant and Mascot/ptmRS (PD) (fully tryptic, 1% FDR).

	**Count at 1% FLR**			**Count at 5% FLR**			**Count at 10% FLR**		
	**pAla**	**pLeu**	**pGly**	**pAla**	**pLeu**	**pGly**	**pAla**	**pLeu**	**pGly**
**TPP**	23104	17943	18872	40514	42157	35939	44556	45875	38885
**PEAKS**	4627	3633	1305	6679	5662	3778	13683	9725	4539
**MaxQuant**	9826	9086	6808	31104	18645	27503	33663	32436	29661
**Mascot/ptmRS**	23646	27294	20976	43840	43918	36414	57305	57591	54720

a)

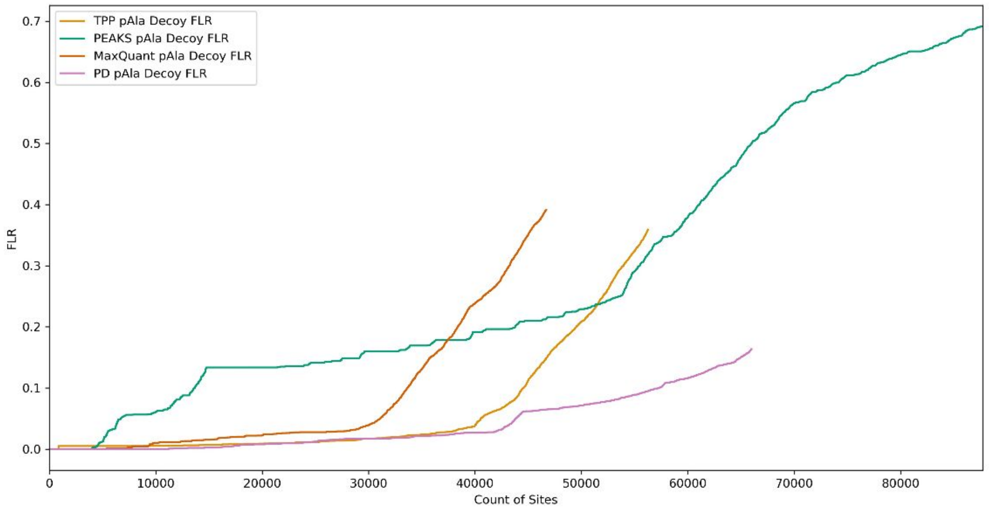

b)

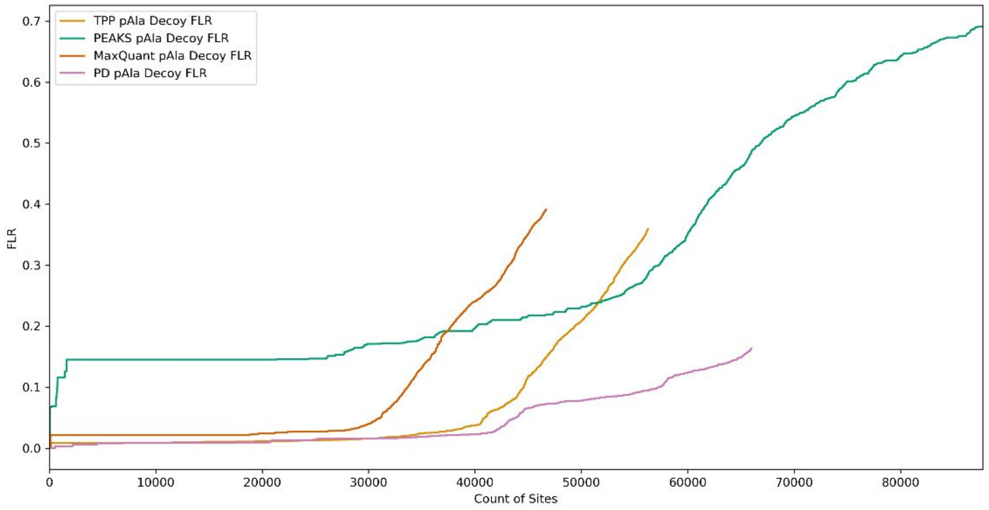

Supplementary Figure 10: Comparison of pAla Decoy FLR estimation searching PXD008355 (Arabidopsis data set) using different pipelines: TPP, PEAKS, MaxQuant, Mascot/ptmRS (PD). a) ordered by combined probability; b) ordered by PTM probability (Fully tryptic, 1% FDR).

Supplementary Table 7: Counts of sites at pAla Decoy FLR for 1%, 5% and 10% using each pipeline, searching PXD008355 (Arabidopsis data set) (fully tryptic, 1% FDR).

	**Ordered by combined probability**			**Ordered by PTM probability**		
**Pipeline**	**Count at 1% FLR**	**Count at 5% FLR**	**Count at 10% FLR**	**Count at 1% FLR**	**Count at 5% FLR**	**Count at 10% FLR**
**TPP**	23104	40514	44556	1468	40921	44576
**PEAKS**	4627	6679	13683	23	23	734
**MaxQuant**	9826	31104	33663	91	31089	33667
**Mascot/ptmRS**	23646	43840	57305	20712	43881	57353

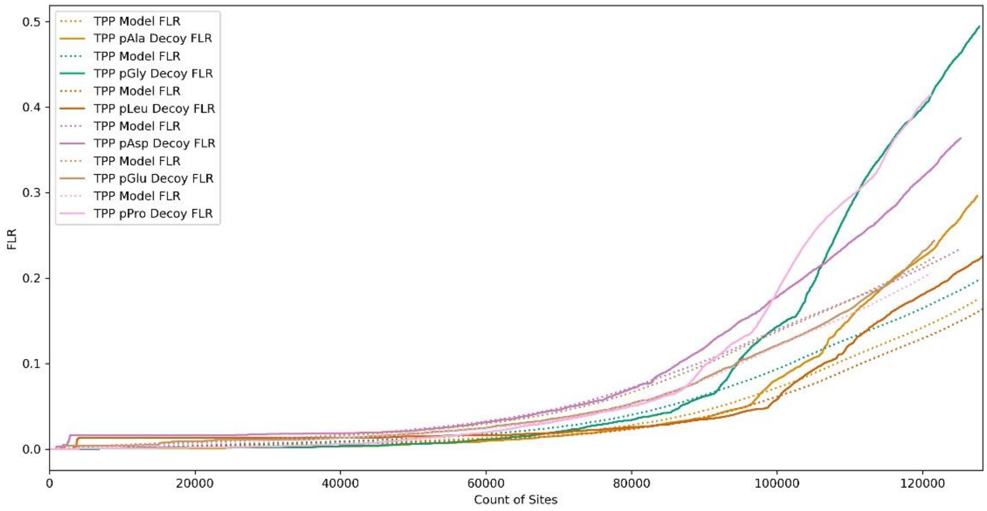

Supplementary Figure 11: Comparison of pX Decoy FLR and Model FLR estimation searching PXD000612 (Human data set) for pAla, pLeu, pGly, pAsp, pGlu and pPro (Fully tryptic, 1 % FDR), using TPP.

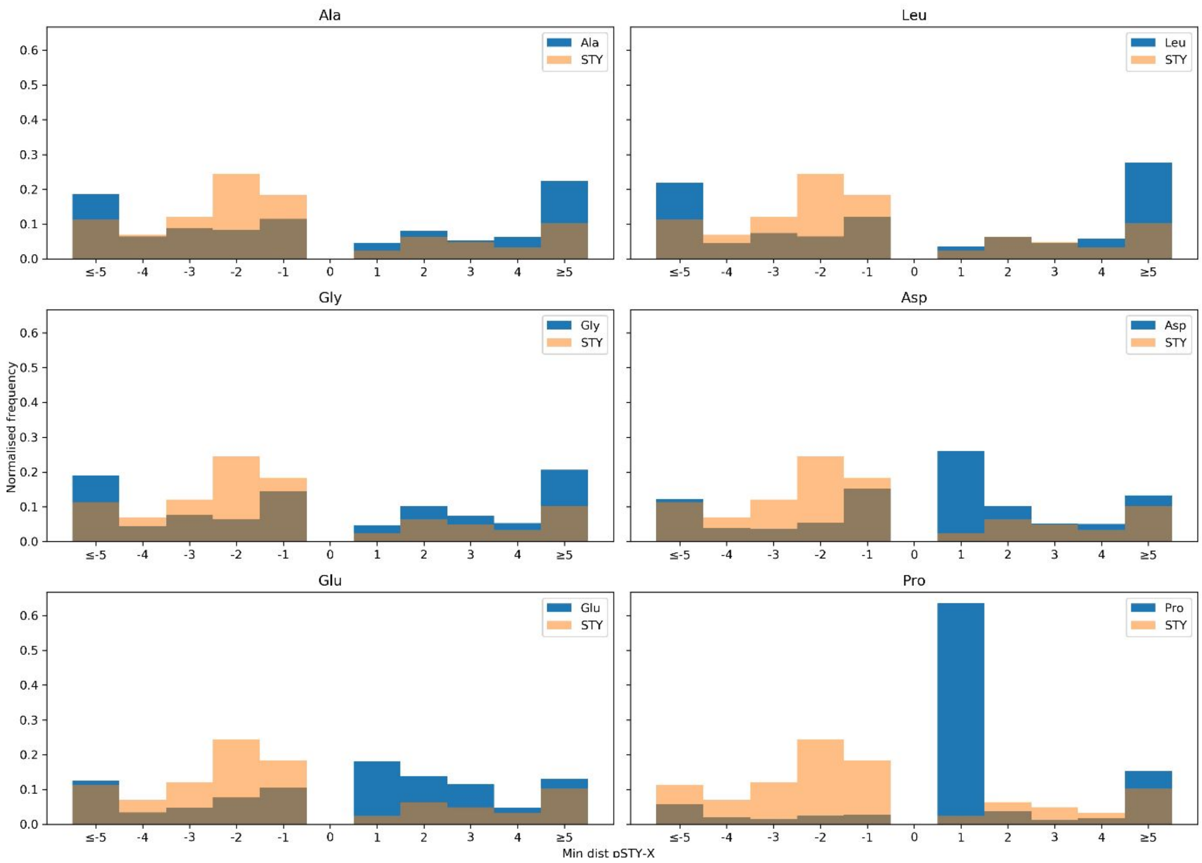

Supplementary Figure 12: Comparison of minimum distance between phosphorylated S, T or Y and the nearest target amino acid (Ala, Leu, Gly, Asp, Glu and Pro), compared to the STY distribution, searching PXD000612 (Human data set).

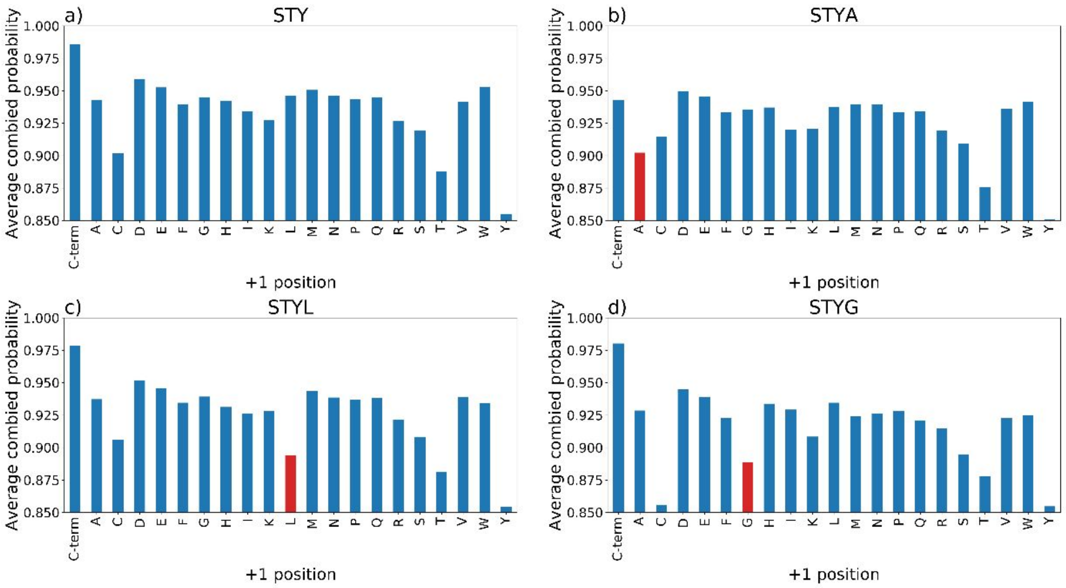

Supplementary Figure 13: Comparison of averaged final site probabilities for all peptides (final probability ≥0.68), split by amino acid in the +1 positions for the PXD008355 (Arabidopsis data set). a) STY (no decoy); b) STY with Ala decoy; c) STY with Leu decoy and d) STY Gly decoy.

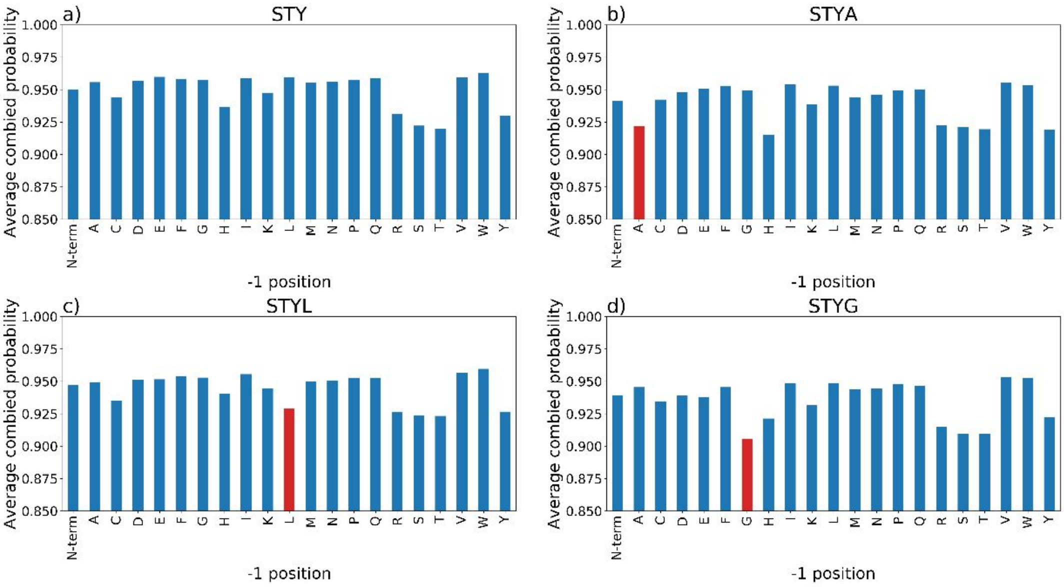

Supplementary Figure 14: Comparison of averaged final site probabilities for all peptides final probability ≥0.68), split by amino acid in the -1 positions for the PXD000612 (Human data set). a) STY (no decoy); b) STY with Ala decoy; c) STY with Leu decoy and d) STY Gly decoy.

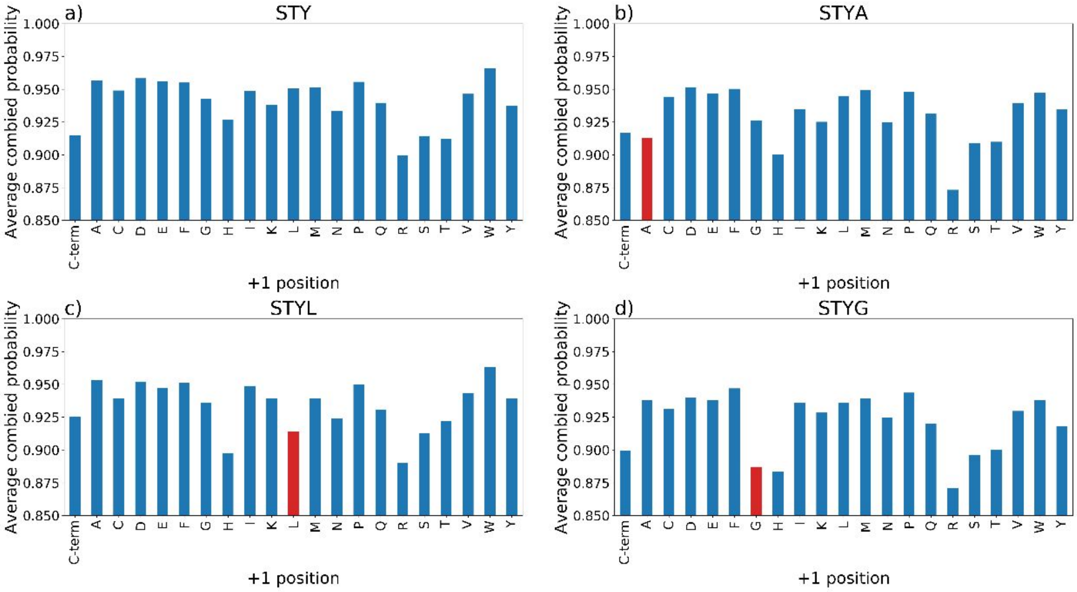

Supplementary Figure 15: Comparison of averaged final site probabilities for all peptides (final probability ≥0.68), split by amino acid in the +1 positions for the PXD000612 (Human data set) a) STY (no decoy); b) STY with Ala decoy; c) STY with Leu decoy and d) STY Gly decoy.
